# Linking mitochondria to the immune response

**DOI:** 10.7554/eLife.56214

**Published:** 2020-04-15

**Authors:** Rebecca L Wallings, Mary K Herrick, Malú Gámez Tansey

**Affiliations:** 1 Department for Neuroscience, University of Florida College of MedicineGainesvilleUnited States; 2 Center for Translational Research in Neurodegenerative Disease, University of Florida College of MedicineGainesvilleUnited States

**Keywords:** Parkinson's disease, cGAS, DRP1, purine biosynthesis, bacterial pathogenesis, metabolism, Human, Mouse, Other

## Abstract

A gene associated with Parkinson’s disease regulates mitochondrial homeostasis, thus affecting innate immunity.

**Related research article** Weindel CG, Bell SL, Vail KJ, West KO, Patrick KL, Watson RO. 2020. LRRK2 maintains mitochondrial homeostasis and regulates innate immune responses to *Mycobacterium tuberculosis*. *eLife* **9**:e51071. doi: 10.7554/eLife.51071

Parkinson’s disease is a motor disorder caused by the loss of a specific sub-set of neurons located in the midbrain and the accumulation of a protein called α-synuclein. The underlying mechanisms that lead to the death of the midbrain neurons are still not well understood. However, many individuals with Parkinson’s disease have increased levels of inflammation in the brain and in peripheral organs, such as the gut. This inflammation is now viewed as a potential contributor to Parkinson’s disease, and not just a result of it.

Mutations in the gene coding for the kinase LRRK2 are the most common genetic cause of inherited Parkinson’s disease (Singleton et al., 2013). Single nucleotide polymorphisms in this gene are also associated with increased susceptibility to inflammatory diseases such as leprosy – which, like tuberculosis, is caused by a mycobacterium – and irritable bowel disease (Fava et al., 2016; Witoelar et al., 2017; Villumsen et al., 2019). Mounting evidence suggests that mutations in LRRK2 contribute to immune alterations in both peripheral organs and the brain (Wallings and Tansey, 2019), but the mechanisms through which LRRK2 regulates these immune responses are poorly understood.

Aside from its link to inflammation, LRRK2 has also been implicated in the activity of mitochondria, which are key subcellular organelles linked to Parkinson’s disease (Singh et al., 2019; Park et al., 2018). For example, neural stem cells derived from the skin of individuals carrying a mutation associated with Parkinson’s disease in the gene for LRRK2 exhibit increased mitochondrial DNA damage as well as increased oxidative damage (Howlett et al., 2017; Sanders et al., 2014). Despite this, the contribution of LRRK2 to mitochondrial health in cells of the peripheral immune system has been understudied. Now, in eLife, Robert Watson and colleagues from Texas A&M University – including Chi Weindel and Samantha Bell as joint first authors – report that LRRK2’s ability to influence inflammatory responses in peripheral immune cells is directly linked to its role in maintaining mitochondrial homeostasis (Weindel et al., 2020).

To investigate the role of LRRK2 in peripheral immune responses, Weindel et al. isolated macrophages from the bone marrow of mice lacking the gene that codes for the mouse homolog of LRRK2 (*Lrrk2* knockout macrophages). Compared to macrophages with a single copy of this gene (*Lrrk2* HETs), the knockout macrophages upregulated genes normally stimulated by interferons (signaling proteins that recruit immune cells to trigger an immune response). Weindel et al. also found that these macrophages were unable to upregulate interferon response genes when they were infected with mycobacteria, likely because these genes were already chronically activated.

Moreover, when mice lacking *Lrrk2* were infected with *Mycobacterium tuberculosis*, which causes tuberculosis, their lungs exhibited exacerbated local inflammation caused by the infection. However, no differences in the outcome of the infection were observed in these mice relative to the *Lrrk2* HETs. This contradicts previous reports suggesting that mice lacking *Lrrk2* can stop *M. tuberculosis* replication more effectively than wild-type mice, correlating with increased inflammation in the lungs (Härtlova et al., 2018). One possible reason for this inconsistency is that Weindel et al. used *Lrrk2* heterozygous controls as opposed to wild-type controls. Furthermore, different *M. tuberculosis* strains were used in the two studies, and there were also differences in the number of bacteria that mice were exposed to during infection.

Since LRRK2 is also involved in mitochondrial activity, Weindel et al. next looked at the mitochondria in *Lrrk2* knockout macrophages. Mitochondria are dynamic organelles that can divide (fragment) or fuse depending on the state of the cell. When a cell is stressed, mitochondria fuse together to exchange damaged DNA and keep aerobic respiration going. Weindel et al. observed that in macrophages lacking *Lrrk2*, mitochondria did not fuse, making the macrophages more susceptible to stress, and leading to mitochondrial DNA leaking into the cytosol. This was due, in part, to increased activation of DRP1, a protein that helps mitochondria fragment. Inhibiting DRP1 successfully rescued these abnormal mitochondria in *Lrrk2* knockout macrophages. Weindel et al. also reported reduced antioxidant levels, a concomitant accumulation of reactive oxygen species, and increased mitochondrial stress in macrophages lacking *Lrrk2*. Treating these macrophages with antioxidants alleviated mitochondrial stress (Figure 1).

**Figure 1.**
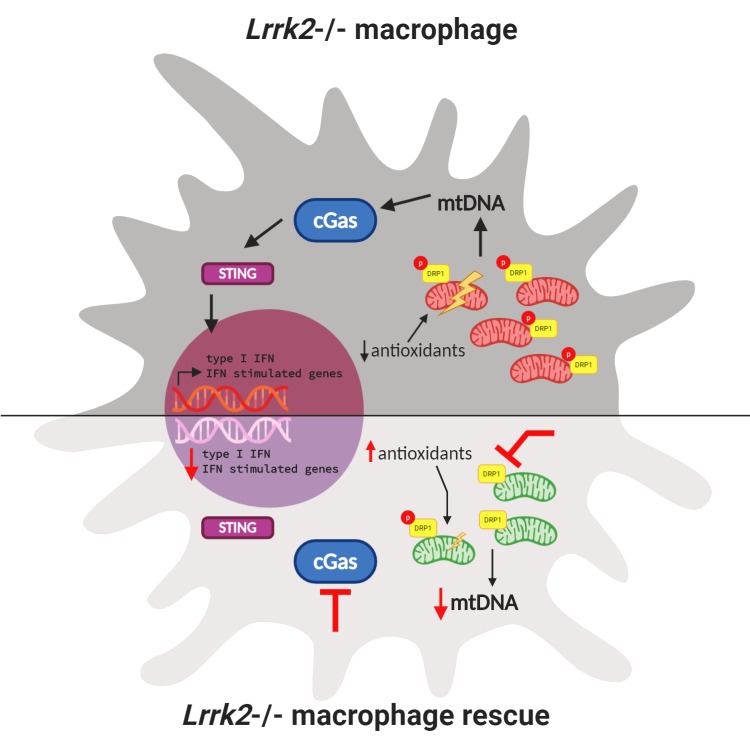
Interactions between mitochondrial homeostasis and immune response in a *Lrrk2* knockout macrophage. (Top) Loss of *Lrrk2* in mouse macrophages increases DRP1 phosphorylation. This leads to an increase in the level of mitochondrial DNA (mtDNA; red bean-shaped structures) in the cytosol, which activates the cGAS/STING pathway. This, in turn, triggers the phosphorylation of transcription factors that activate the expression of interferon (IFN) response genes in the nucleus, which leads to a lowered response to interferon from these cells. In this situation, antioxidant levels in the cell are low and mitochondrial stress increases. (Bottom) Three different treatments can partially or totally rescue inflammatory deficits in *Lrrk2* knockout macrophages. First, treating these macrophages with antioxidants alleviates mitochondrial stress and rescues the normal response to interferon. Second, inhibiting DRP1 phosphorylation decreases fission, lowering the level of mitochondrial DNA (green bean-shaped structures) in the cytosol. This prevents the activation of the cGAS/STING pathway, and consequently, the abnormal activation of interferon response genes. Third (and confirming this finding), removing the *cGas* gene also mitigates increased interferon stimulated gene expression.

The increased mitochondrial DNA in the cytosol of *Lrrk2* knockout macrophages led Weindel et al. to hypothesize that the inability to regulate mitochondrial homeostasis could be contributing to chronic activation of interferon response genes. The cGAS–STING pathway is a part of the innate immune system that triggers the expression of inflammatory genes when DNA is detected in the cytosol. Weindel et al. showed that loss of the *cGas* gene decreased basal levels of type I interferon in *Lrrk2* knockout macrophages and corrected the response to immune stimuli (Figure 1).

These findings link the role of LRRK2 in innate immune dysregulation with its critical function in maintaining mitochondrial homeostasis for the first time and have wider implications for the field of Parkinson’s disease. Future research will likely investigate how mutations in the gene for LRRK2 affect the relationship between mitochondria and inflammation in immune cells. The most common of these mutations causes a toxic increase in kinase activity, so kinase inhibitors have been considered for their potential therapeutic effects in Parkinson’s disease. However, given that loss of LRRK2 may lead to higher risks of infection and inflammation in peripheral blood cells, therapeutic windows will have to be carefully monitored to avoid unwanted effects on the immune system.
